# Relationship between discordant response to HAART, Tregs, immune activation and low-level viraemia

**DOI:** 10.7448/IAS.17.4.19672

**Published:** 2014-11-02

**Authors:** Julien Saison, Tristan Ferry, Julie Demaret, Delphine Maucort-Boulch, Fabienne Venet, Thomas Perpoint, Florence Ader, Vinca Icard, Christian Chidiac, Guillaume Monneret

**Affiliations:** 1 Infectious and Tropical Disease Unit, Hôpital de la Croix-Rousse, Lyon, France; 2 Immunology Laboratory, Hôpital Edouard Herriot, Lyon, France; 3 Biostatistic Unit, Hospices Civils de Lyon, Lyon, France; 4 Immunology Laboratory, Hopital Edouard Herriot, Lyon, France

## Abstract

**Introduction:**

The incomplete immune recovery upon effective long-term highly active antiretroviral therapy (HAART) has been associated with increased morbidity and mortality in HIV infected patients [1]. Immune cellular activation, Tregs or very low-level viraemia has been alternatively suspected, but never investigated simultaneously [2].

**Materials and Methods:**

We performed a cross-sectional study in 87 aviraemic patients (men=62, mean CD4+T cells=570/mm^3^, mean duration of HAART=12 years). Patients with at least 500 CD4+ T cells /mm^3^ were classified as complete immunological responders (cIR), whereas remaining patients were classified as inadequate immunological responder (iIR). Tregs were characterized based on CD4+CD25highFoxP3+phenotype using a one-step intracellular staining. Effector Tregs and terminal effectors Tregs were respectively defined as CD4+CD25+FoxP3+CD45RA-, and CD4+CD25+FoxP3+CD45RA-HLADR+phenotypes as recently described [3]. Activated T cells were identified using (i) elevated HLA-DR expression for CD4+T cells, and (ii) increased expressions of HLA-DR, or CD38, or both (HLADR+CD38+cells) for CD8+T cells. Very low-level viraemia was defined as detectable viraemia between 1 and 39 cp/mL. Univariate and multivariate analyses were performed to identify determinants of iIR.

**Results:**

Thirty-nine patients were classified as iIR, and 48 as cIR. Patients from the iIR group were significantly older (55 vs 50 years, p=0.027), and had percentages of activated CD4+ T cells, Tregs, effector Tregs and terminal effector Tregs significantly higher (5.3 vs 4%, p=0.014; 9 vs 7.5%, p=0,022; 8 vs 6.3%, p=0.01 and 1.8 vs 1.3%, p=0,033 among CD4+T cells, respectively). Neither the percentage of activated CD8+T cell nor very low-level viraemia were found to be associated with iIR. In the multivariate analysis, nadir of CD4+T cell count and percentage of Tregs were the only two parameters independently associated with iIR (OR=2.339, p=0.001, and OR=0.803, p=0.041, respectively).

**Conclusions:**

We present here the largest study investigating simultaneously immune response to long-term HAART, immune activation of CD4+ and CD8+ T cells, Tregs percentages and very low-level viraemia. Our results highlight the importance of Tregs in CD4 homeostasis. This aspect should now be prospectively explored in a large cohort of patients.

## References

[1] Rodger AJ, Lodwick R, Schechter M, Deeks S, Amin J, Gilson R (2013). Mortality in well controlled HIV in the continuous antiretroviral therapy arms of the SMART and ESPRIT trials compared with the general population. AIDS.

[2] Gaardbo JC, Hartling HJ, Gerstoft J, Nielsen SD (2012). Incomplete immune recovery in HIV infection: mechanisms, relevance for clinical care, and possible solutions. Clin Dev Immunol.

[3] Sakaguchi S, Miyara M, Costantino CM, Hafler DA (2010). FOXP3+ regulatory T cells in the human immune system. Nat Rev Immunol.

